# Analysis of molecular diversity within single cyanobacterial colonies from environmental samples

**DOI:** 10.1038/s41598-020-75303-2

**Published:** 2020-10-28

**Authors:** M. Ángeles Muñoz-Martín, Esther Berrendero Gómez, Elvira Perona, Pilar Mateo

**Affiliations:** 1 Departamento de Biología, Facultad de Ciencias, Universidad Autónoma de Madrid, 28049 Madrid, Spain; 2 Departamento de Biología Aplicada, Facultad de Ciencias Experimentales, Universidad de Miguel Hernandez, 03202 Elche, Spain

**Keywords:** Ecology, Microbiology, Environmental sciences

## Abstract

Attached or floating macroscopic cyanobacteria can be found in shallow waters and can be easily hand-collected, but their identification is often challenging due to their high morphological variability. In addition, many members of environmental samples lose their morphological adaptations under controlled conditions, making the integration of analyses of field populations and derived isolated cultures necessary in order to evaluate phenotypic plasticity for identification purposes. Therefore, in this study, twenty-nine macroscopic field samples were analyzed by Illumina sequencing and parallel optical microscopy. Some colonies showed the typical morphological characteristics of *Rivularia biasolettiana*, and others showed those of *Rivularia haematites*. However, other *Rivularia*-like colonies showed ambiguous morphologies, and some of them showed the phenotypic features of the new genus *Cyanomargarita*, which is virtually indistinguishable from *Rivularia* in the field. In all of the colonies, phylotype composition was highly heterogeneous, with abundances varying depending on the analyzed sample. Some colonies were dominated (97–99%) by a single phylotype, while in others, the percentage of the dominant phylotype decreased to approximately 50–60%. Surprisingly, the same dominant phylotype was found in *R. biasolettiana* and *R. haematites* colonies. The relationships between environmental and/or biological factors and morphological variability in these colonies are discussed.

## Introduction

Cyanobacteria are a very special group of gram-negative prokaryotes. Because they developed the ability to use water as an electron donor in oxygenic photosynthesis, approximately 3.6 billion years ago^1^, they played an important role in the evolution of life on Earth, the subsequent generation of molecular O_2,_ and the oxygenation of the atmosphere^2^. Cyanobacteria are also the ancestors of chloroplasts^3,4^. In addition, many cyanobacteria are capable of fixing atmospheric N_2_ and thus play an important role in nitrogen cycling^5^.

It is easy to understand why cyanobacteria were previously called blue-green algae since, in addition to performing plant-like oxygenic photosynthesis, macroscopic green forms similar to algae can be easily found and observed by the naked eye, with a cosmopolitan distribution ranging from hot hyperarid deserts to polar aquatic environments^6^. These alga-like forms, in which individuals are microscopic but exhibit macroscopic growth, have been described as colonies, thalli, mats, tufts, thin coatings, soft gelatinous covers, subspherical globes, tightly attached felts, etc.^7–9^.

The importance of characterizing and identifying natural cyanobacterial populations in order to compare them with corresponding cultures, which are further used, e.g., in phylogenetic analysis or potential applications in biotechnology, has been emphasized^10–13^. However, unfortunately, the majority of recent studies were based only on isolated strains and morphological descriptions corresponding to culture media, and controlled conditions were often different from the characteristics found in the environments where highly diverse cyanobacteria live^7,13^. In a previous study, we compared phenotypic characteristics of natural populations and isolated cultures from environmental samples and found large differences between them, which can lead to misidentification of strains^12^.

Rivulariaceae includes all the heterocystous cyanobacteria with tapered trichomes, with a clear distinction from the base to the apex. A terminal heterocyst (heterocyte) occurs at the broad basal end of the trichomes, and a sheath encloses everything but the heterocyst^7^. These tapering cyanobacteria include many visually conspicuous morphotypes found widely in different aquatic environments; for instance, *Rivularia*, *Dichothrix* and *Gloeotrichia* form macroscopic attached or floating colonies in shallow waters, and many species of *Calothrix* also form a macroscopic thallus with a characteristic appearance^14^.

*Rivularia* colonies are hemispherical or almost spherical, with more or less parallel sheathed trichomes arranged radially inside colonies; the trichomes often have false branches. *Dichothrix* forms small colonies of various shapes, mostly cushions or dense tufts; the trichomes are subdichotomic and falsely branched, and there are often many trichomes inside one sheath. *Calothrix* includes all the forms growing as individual filaments or ill-defined colonies. Filaments in *Gloeotrichia* colonies are easy to distinguish by their ability to form akinetes^7,14^.

Recently, a new genus of tapering heterocystous cyanobacteria, *Cyanomargarita*, was described^15^. Natural populations were completely consistent with the description of *Rivularia*; however, upon sequencing, they were found to be phylogenetically distant from *Rivularia*. In addition, several other new genera of tapering cyanobacteria have been described, such as *Macrochaete*^16^ and *Dulcicalothrix*^17^, but these were based only on isolated cultured strains; therefore, the characteristics of natural populations are unknown.

Nevertheless, assessments of conspicuous natural populations have suggested the heterogeneity of colonies^14^. A previous analysis indicated that *Rivularia* colonies were heterogeneous, where three different morphological types of tapering trichomes were isolated from a single colony of *Rivularia*^18^, and our previous studies combining morphological characterization and genetic characterization via 16S rRNA and phycocyanin operon genes in *Rivularia* colonies suggested genotypic diversity within a single colony^11^.

To study the variability and proportions of genotypes within single colonies, 28 *Rivularia-*like colonies were collected and analyzed by Illumina sequencing (16S rRNA gene) and parallel optical microscopy. In addition, other type of macroscopic growth not showing the typical hemispherical shape of *Rivularia* colonies but rather a brush-like *Dichothrix* tuft was also assessed for comparison with other Rivulariaceae.

Since the occurrence of *Rivularia* colonies is related to oligotrophic, high-altitude mountain areas and/or clean calcareous running waters^14^, where they carry out activities such as N_2_ fixation^19^ and phosphatase activity^20^ in response to the low levels of combined N and P, respectively, different oligotrophic systems, with previously studied *Rivularia* colonies^11,19–23^, in a latitudinal gradient in Spain, were selected. In addition, another location in northern England, where *Rivularia* colonies have long occurred and been analyzed^24–26^ was chosen to assess possible geographic variations.

## Results

### Morphological characterization of environmental samples

**Figure 1:**
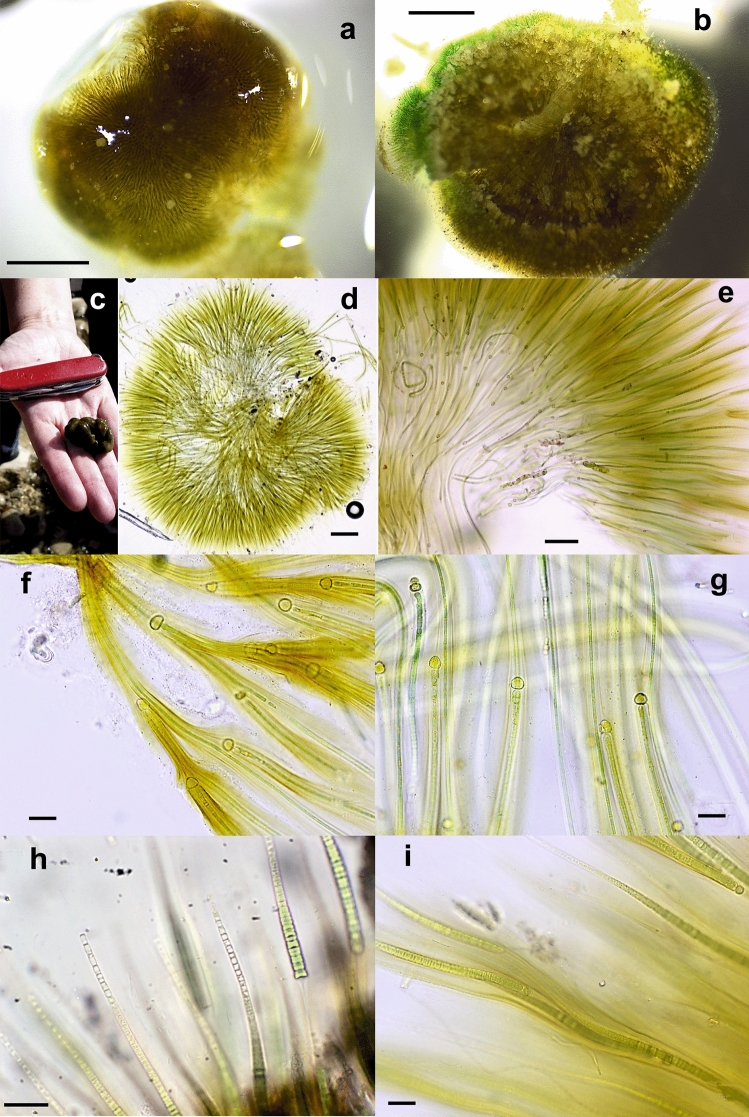
Photographs and light micrographs of *Rivularia biasolettiana*-type colonies. (**a**) BAT5 and (**b**) BAT14 colonies from the Bogarra River. (**c**) GG2 colony from the Guarga River. (**d**) Radial arrangement of filaments in the BAT5 colony from the Bogarra River. (**e**) Detail of the radial arrangement of filaments in the END5 colony from the Endrinales River. (**f**) and (**g**) BAT12 filaments showing pigmented or hyaline sheaths. (**h**) Hairs in filaments of the GG2 colony from the Guarga River. (**i**) Meristematic zones showing divided trichomes persisting within common old sheaths in END5 from the Endrinales River. Bars 1 mm (**a**,**b**), 200 μm (**d**), 100 μm (**e**), 20 μm (**f**–**i**).

**Figure 2:**
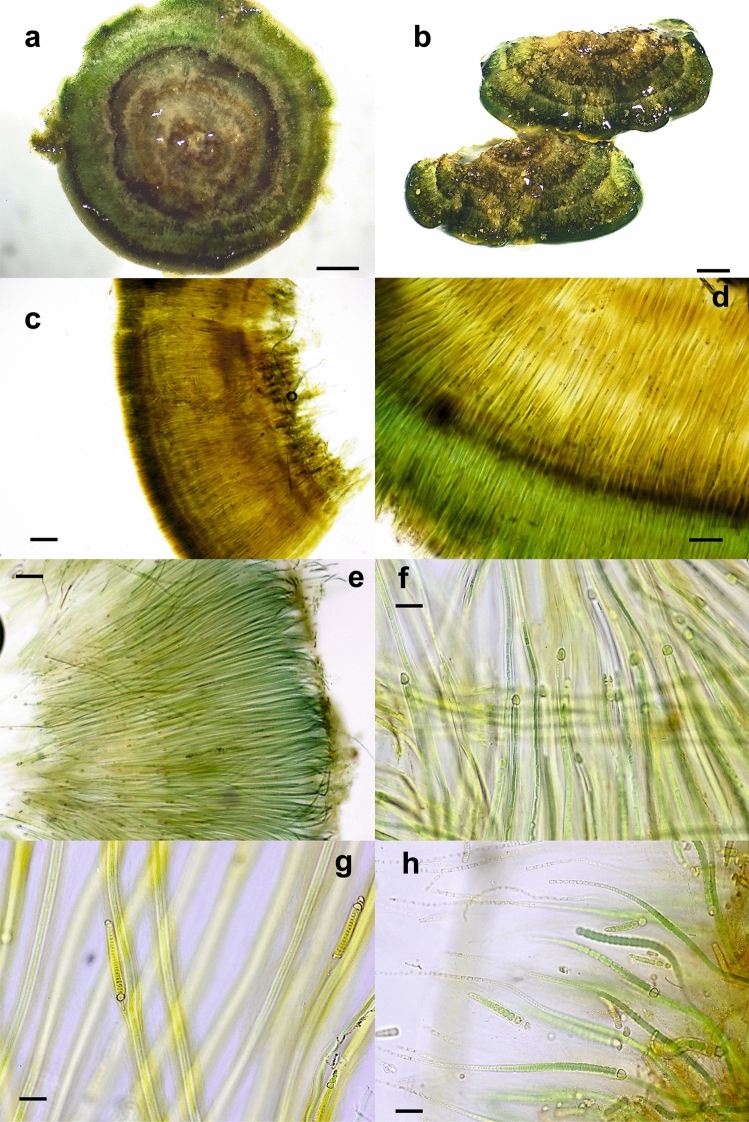
Light micrographs of *Rivularia haematites-*type colonies. (**a**) HOY14 and (**b**) HOY13 colonies from the Hoyas stream, showing high calcification and concentric zonation. Sections of END15 (**c**) and END2 (**d**) colonies from the Endrinales stream, showing zonation after decalcification with EDTA corresponding to layers of calcite, still obvious due to differences in sheath density and scytonemin pigmentation. Parallel and densely arranged filaments in the GOR4 (**e**) and GOR5 (**f**) colonies from Gordale Beck. (**g**) Hairs of trichomes of the HOY3 colony from the Hoyas stream. (**h**) New trichomes persisting within common old sheaths in the END15 colony from the Endrinales stream. Bars 1 mm (**a**,**b**), 200 μm (**c**), 100 μm (**d**), 20 μm (**e**–**h**).

**Figure 3:**
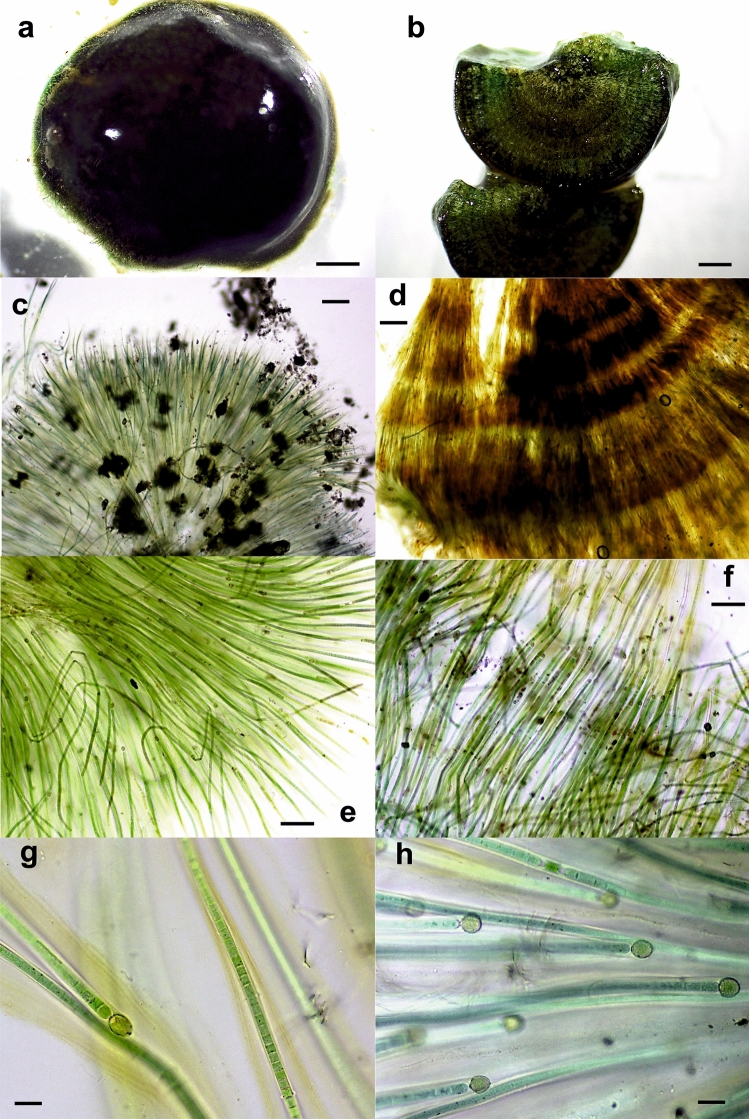
Light micrographs of *Cyanomargarita* colonies. (**a**) Hemispherical END1 colony from the Endrinales stream. (**b**) Section of the END1 colony showing zonation. (**c**) Radial arrangement of filaments in the ARA4 colony from the Aras River. (**d**) Layers with distinct pigmentation in the decalcified END8 colony from the Endrinales stream. (**e**) Tapering trichomes in the END8 colony from the Endrinales stream, similar to those of *Rivularia*. (**f**) Parallel and densely arranged filaments in the END1 colony from the Endrinales stream. Colored and lamellated sheaths of filaments in the ARA4 colony from the Aras River and (**g**) hyaline sheaths in other parts of the same ARA4 colony (**h**). Bars 1 mm (**a**,**b**), 200 μm (**c**,**d**), 100 μm (**e**,**f**), 20 μm (**g**,**h**).

**Figure 4:**
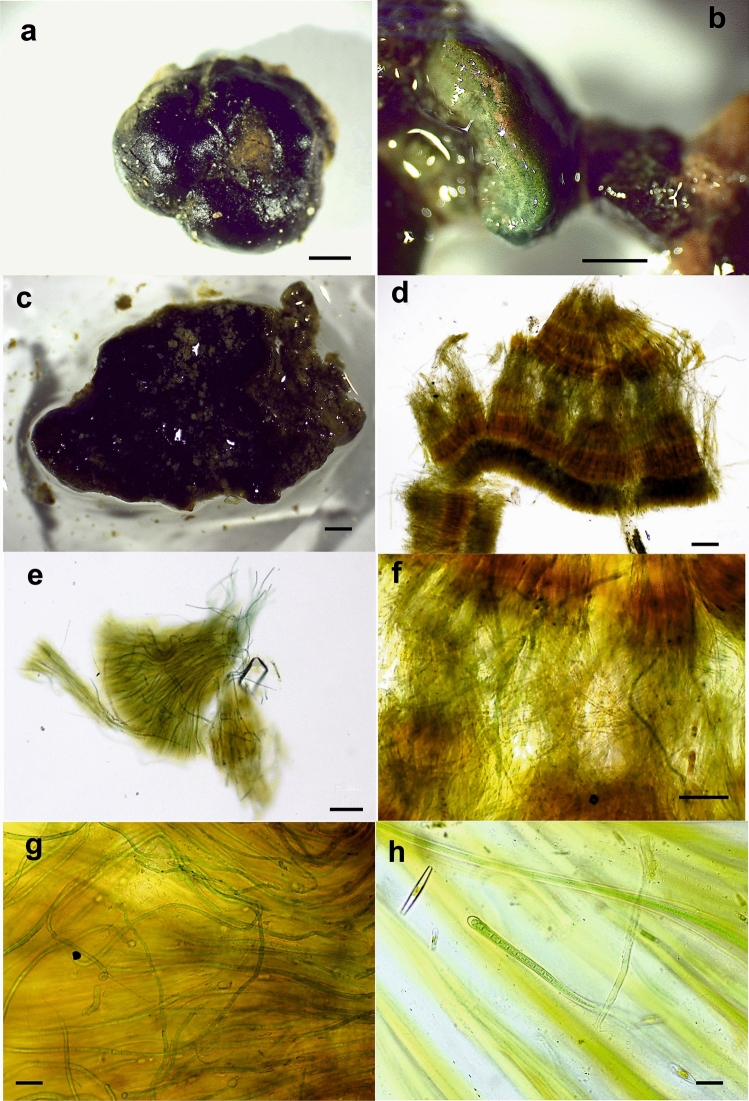
Light micrographs of colonies with ambiguous morphology. (**a**) Calcified hemispherical GDL1 colony from the Guadiela River. (**b**) Section of the MU4 colony from the Muga River showing layers. (**c**) Slightly irregular-hemispherical and calcified BAT4 colony from the Bogarra River. (**d**) Zonation with distinct pigmentation in the decalcified BAT4 colony. (**e**) Large proportion of isopolar filaments without heterocysts within the GDL1 colony. (**f**) Heterogeneity in the trichomes found in the BAT4 colony. (**g**) Detail of isopolar filaments without heterocysts within the GDL1 colony. (**h**) Calothrix filament found in the BAT2 colony from the Bogarra River. Bars, 1 mm (**a**–**c**), 200 μm (**d**), 100 μm (**e**,**f**), 20 μm (**g**,**h**).

**Figure 5:**
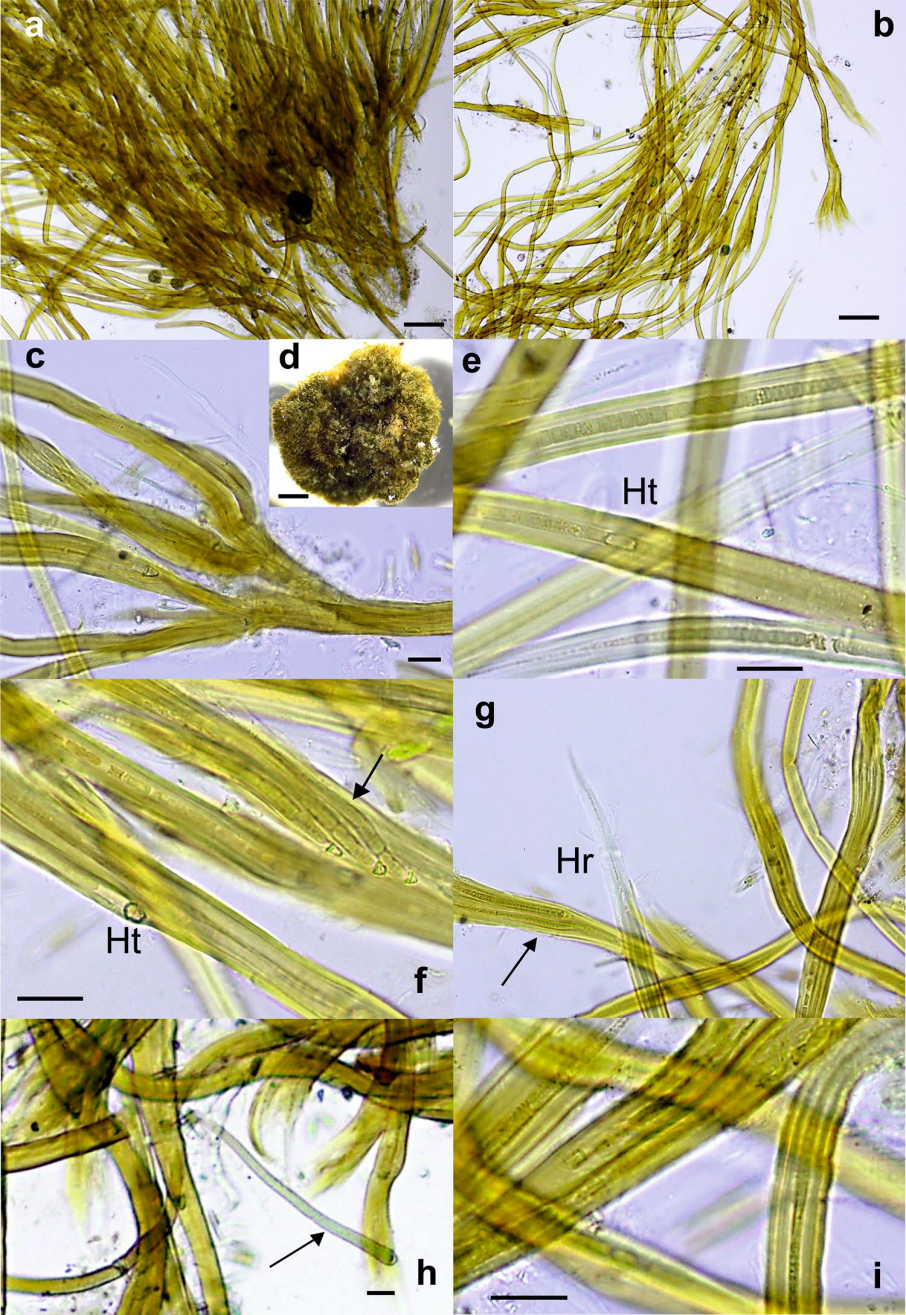
Light micrographs of the BAT13 colony from the Bogarra River. (**a**) Calcified hemispherical colony. (**b**) Dichotomic and radial arrangement of filaments. (**c**) Bunch of trichomes, some of them showing a funnel-like end. (**d**) Dichotomic arrangement with false branches. (**e**) Sheaths distinctly broader than the trichome, showing an intercalary heterocyst. (**f**) Secondary trichomes remaining within the ‘mother’ sheath. In a filament, three trichomes in a common sheath (arrow). (**g**) Thick yellow–brown sheath with a trichome tapering into a hyaline hair. (**h**) Filament of *Calothrix* found inside the sample. (**i**) Lamellated sheaths. Bars 1 mm (**a**), 100 μm (**b**,**c**), 20 μm (**d**–**i**).

Twenty-eight collected samples (Table 1) showed a macroscopic *Rivularia*-like morphology in which hemispherical or slightly irregular- hemispherical lobate colonies approximately 0.5–3 cm in diameter/length could be observed (Figs. 1, 2, 3, 4 and 5). After microscopic evaluation, the *Rivularia*-like colonies could be separated according to their specific features.

**Table 1:** Macroscopic environmental samples analyzed in this study.

Environmental sample	Geographical origin
MUD1 tuft	Muga River, Pyrenees, Girona, north-east Spain
MU4 colony	Muga River, Pyrenees, Girona, north-east Spain
MU5 colony	Muga River, Pyrenees, Girona, north-east Spain
GG1 colony	Guarga River, Pre-Pyrenees, Huesca, north-east Spain
GG2 colony	Guarga River, Pre-Pyrenees, Huesca, north-east Spain
OSI3 colony	Osia River, Pyrenees, Huesca, north-east Spain
ARA4 colony	Aras River, Pyrenees, Huesca, north-east Spain
GDL1 colony	Guadiela River, Hoz de Beteta, Cuenca, central Spain
GOR1 colony	Gordale Beck, West Yorkshire, northern England, UK
GOR2 colony	Gordale Beck, West Yorkshire, northern England, UK
GOR3 colony	Gordale Beck, West Yorkshire, northern England, UK
GOR4 colony	Gordale Beck, West Yorkshire, northern England, UK
GOR5 colony	Gordale Beck, West Yorkshire, northern England, UK
GOR11 colony	Gordale Beck, West Yorkshire, northern England, UK
GOR12 colony	Gordale Beck, West Yorkshire, northern England, UK
HOY3 colony	Hoyas stream, Paterna del Madera, Albacete, south-east Spain
HOY5 colony	Hoyas stream, Paterna del Madera, Albacete, south-east Spain
HOY14 colony	Hoyas stream, Paterna del Madera, Albacete, south-east Spain
BAT2 colony	Bogarra River, Batán de Bogarra, Albacete, south-east Spain
BAT4 colony	Bogarra River, Batán de Bogarra, Albacete, south-east Spain
BAT5 colony	Bogarra River, Batán de Bogarra, Albacete, south-east Spain
BAT12 colony	Bogarra River, Batán de Bogarra, Albacete, south-east Spain
BAT13 colony	Bogarra River, Batán de Bogarra, Albacete, south-east Spain
BAT14 colony	Bogarra River, Batán de Bogarra, Albacete, south-east Spain
END1 colony	Endrinales stream, Espineras, Albacete, south-east Spain
END2 colony	Endrinales stream, Espineras, Albacete, south-east Spain
END5 colony	Endrinales stream, Espineras, Albacete, south-east Spain
END8 colony	Endrinales stream, Espineras, Albacete, south-east Spain
END15 colony	Endrinales stream, Espineras, Albacete, south-east Spain

Nine colonies (BAT5, BAT12, BAT14, END5, GG1, GG2, MU5, GOR1 and GOR3) showed the typical characteristics of *R. biasolettiana*^7^, such as soft gelatinous colonies, an easily crushed structure, and occasional encrusting by calcareous particles (Fig. 1a–c). The trichomes gradually narrowed (Fig. 1d–i), elongating into long hyaline hairs (Fig. 1h,i). In meristematic zones, divided trichomes persisted within common old sheaths (Fig. 1f–i). Seven colonies (GOR4, GOR5, END2, END15, HOY3, HOY5 and HOY14) corresponded clearly to *R. haematites*^7^, with highly calcified and hard hemispherical colonies, with concentric layers (Fig. 2a,b) displaying parallel filaments that were radially and densely arranged (Fig. 2c–f) and sections showing obvious zonation (Fig. 2c,d). As in *R. biasolettiana*, the trichomes gradually narrowed, ending in hyaline hairs (Fig. 2e–g), and new trichomes persisted within common sheaths (Fig. 2h). The dimensions of trichomes, filaments and heterocysts are shown in Table 2. Although some colonies of *R. haematites* showed slightly wider cells than those found in *R. biasolettiana* colonies, with greater variability (Table 2), the mean trichome diameters, which ranged from 3.9–5.6 μm in *R. haematites* and 4–4.7 μm in *R. biasolettiana*, did not show significant differences (*p* > 0.05).

**Table 2:**
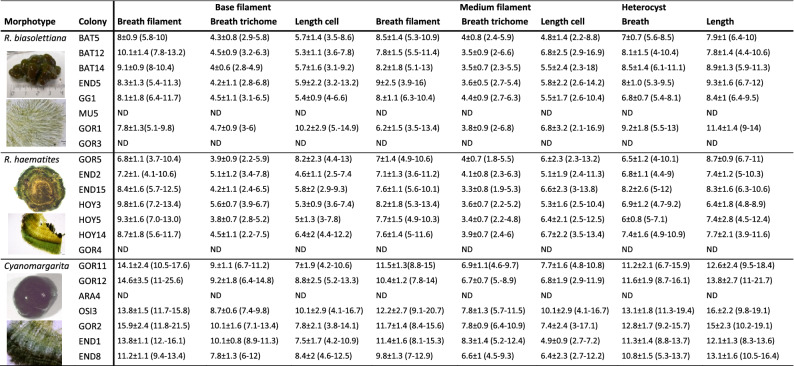
Morphological characteristics of *Rivularia*-like colonies with a dominant morphotype.

Measurements are given as mean ± standard deviation (range) in μm. ND: Not Determined.

Seven other colonies (GOR11, GOR12, OSI3, ARA4, GOR2, END1 and END8) were also hemispherical, some of them soft and gelatinous and others highly calcified with sections showing zonation (Fig. 3a–d). The trichomes also showed the typical tapering of those in *Rivularia* colonies and other similar morphological characteristics (Fig. 3a–h). However, the main difference between these colonies and the previously analyzed colonies was the size of the cells (Table 2, Fig. 3g,h), which were clearly wider in these colonies (significant differences, *p* < 0.05). The mean trichome diameter at the filament base ranged from 7.8 to 10.1 μm. The heterocysts were also clearly larger (Table 2). These features corresponded with those of the new genus *Cyanomargarita*^15^***.***

However, four *Rivularia*-like colonies (MU4, GDL1, BAT2 and BAT4) showed ambiguous morphology, varying in the degree of calcification and zonation (Fig. 4a–d) as well as displaying high heterogeneity in the trichomes (Fig. 4e–h). For instance, GDL1 was a highly calcified colony with a certain degree of zonation, and most of the filaments were of the *Rivularia* type, but there was a considerable proportion of other morphotypes within the colony, such as thinner and nontapered trichomes, without heterocysts, that were found between the *Rivularia* filaments and sometimes emerging outside the colony (Fig. 4a,e,g). The MU4, BAT2 and BAT4 colonies also presented filaments and trichomes with very different sizes and shapes in the same colony (Fig. 4b–d,f,h). In BAT4, clear zonation could be observed (Fig. 4d,f), as well as a large number of filaments without heterocysts (Fig. 4f).

Microscopic examination of the hemispherical and calcified BAT13 colony (Fig. 5a) showed features different from those of the previously analyzed colonies (Fig. 5b–i). Most of the filaments did not taper, although this was due to the presence of a wide and colored sheath (Fig. 5b–i). When observed in detail, the trichomes narrowed from the base, which presented a heterocyst, to the apex (Fig. 5c–g). Although this colony appeared macroscopically as a *Rivularia* colony (Fig. 5a), the trichome arrangement resembled that in the genus *Dichothrix*^7^, such as subdichotomic falsely branched filaments, where secondary trichomes remained within the ‘mother’ sheath (Fig. 5c,d,f). Some filaments showed a bunch of trichomes, open at the end with a funnel-like ending (Fig. 5c). The sheaths were distinctly broader than the trichomes (Fig. 5d–i), often lamellated (Fig. 5e,i), and frayed but closed at the ends (Fig. 5g). The trichomes displayed thin cells in the thick yellow–brown sheath that were quadratic or shorter than wide at the end, tapering into a hyaline hair (Fig. 5e–g). The heterocysts were basal and mainly conical (Fig. 5d,f), but some intercalary and cylindrical heterocysts could be observed (Fig. 5e). Filaments of *Calothrix* were also found inside the sample (Fig. 5h).

**Figure 6:**
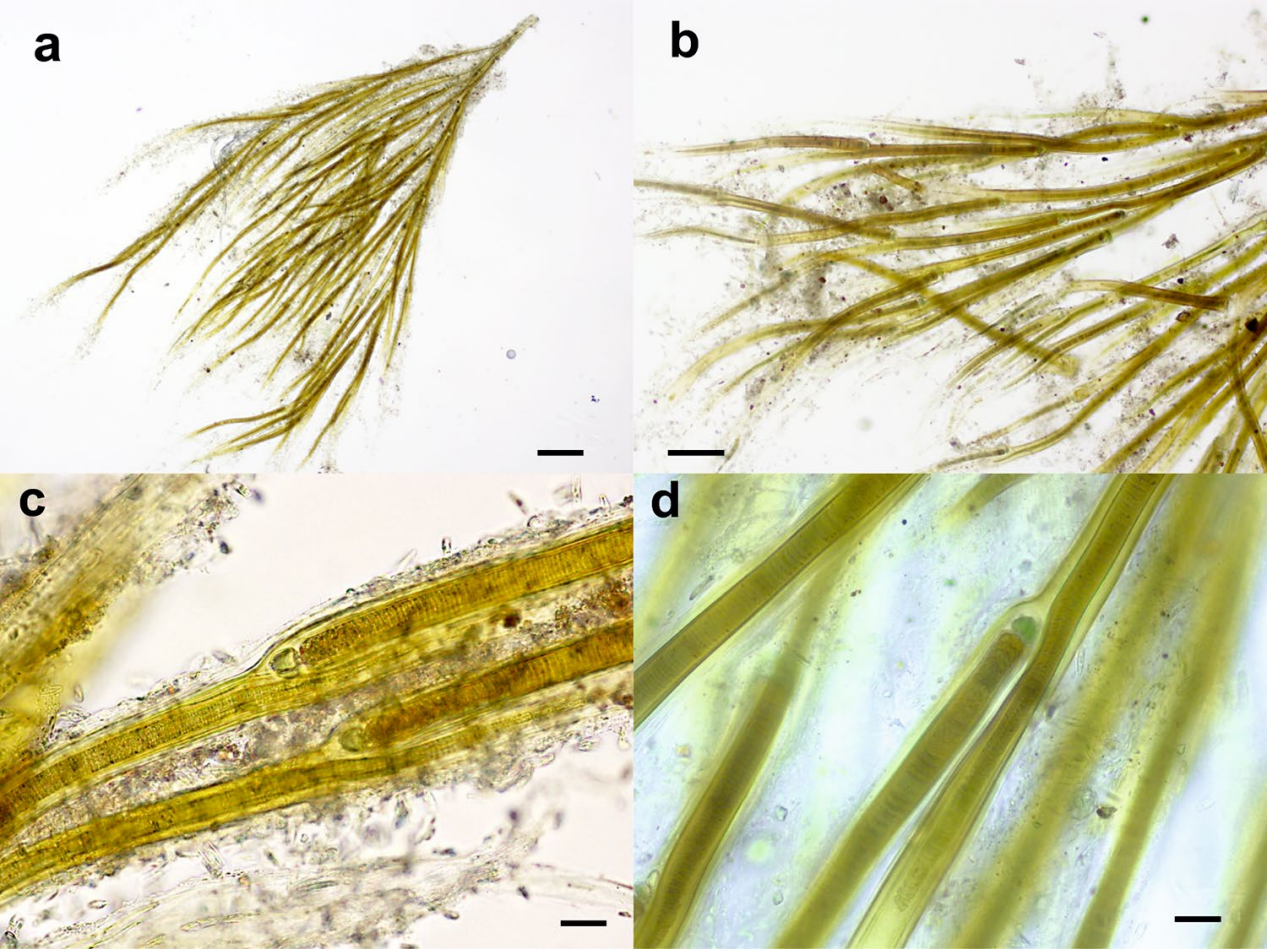
Light micrographs of a *Dichothrix* tuft from the Muga River. (**a**) Brush-like fasciculated MUD1 tuft encrusted by calcareous precipitate. (**b**) Dichotomic arrangement with repeated false branches. (**c**) Lateral false branching adjacent to heterocysts. (**d**) Decalcified filaments showing a new trichome sharing the sheath with the old trichome in the basal part. Bars 200 μm (**a**), 100 μm (**b**), 20 μm (**c**,**d**).

Finally, sample MUD1 showed the typical characteristics of the genus *Dichothrix*^7^, such as macroscopic brush-like fasciculated tufts encrusted by calcareous precipitate (Fig. 6a–d) and filaments in a characteristic dichotomic arrangement that were repeatedly falsely branched (Fig. 6a,b). The filaments were tapered and had strong lateral false branching adjacent to heterocysts (Fig. 6c,d). The branches formed individual sheaths and diverged from the basal filament in parallel, and the new trichome shared the sheath with the old trichome in the basal part (Fig. 6c,d). The sheaths were firm, yellow to brown, and lamellated in only some filaments (Fig. 6d). The trichomes gradually narrowed towards the apex, with barrel-shaped cells (Fig. 6c,d). No hairs were observed. The basal heterocysts were hemispherical or barrel-shaped with a characteristic intense blue-green color (Fig. 6d).

### Cyanobacterial taxonomic assignments

**Figure 7:**
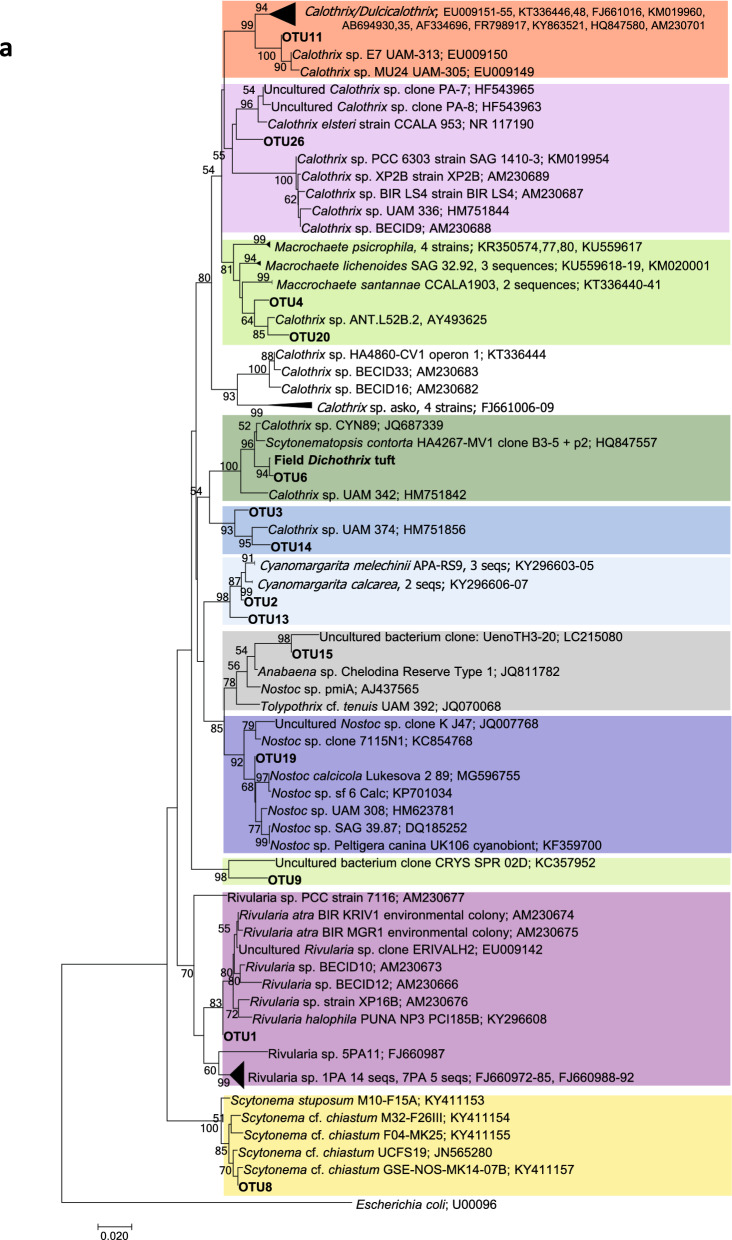

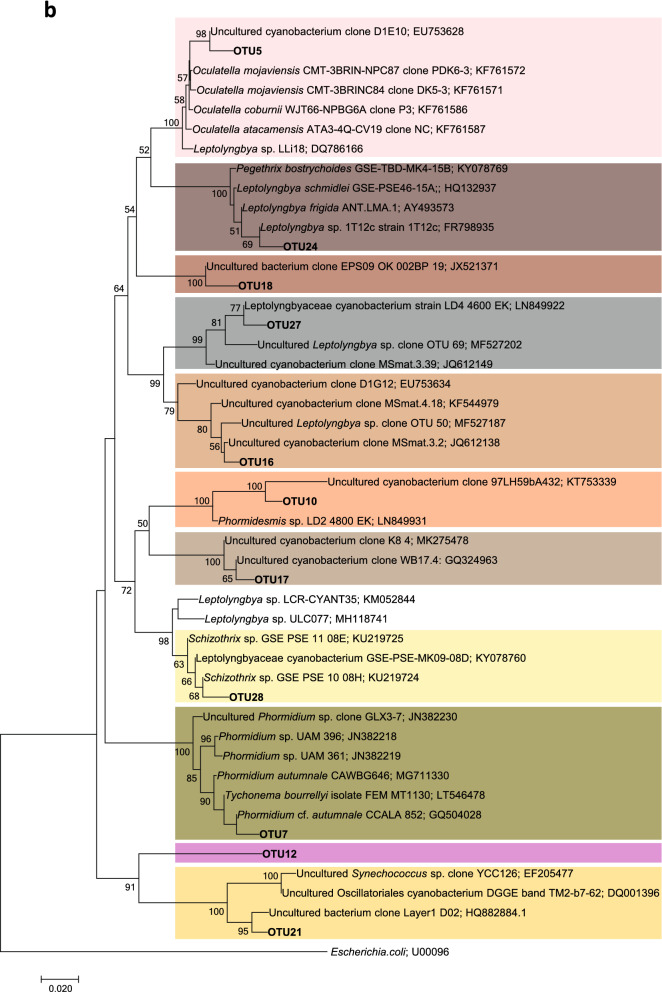
Phylogenetic trees obtained by the neighbor-joining method representing (**a**) heterocystous cyanobacteria and (**b**) filamentous nonheterocystous cyanobacteria, based on the analysis of the 16S rRNA gene, showing the position of the operational taxonomic units (OTUs) and the sequence obtained by cloning a *Dichothrix* environmental sample obtained from the present study (in bold). Numbers near nodes indicate bootstrap values greater than or equal to 50.

The results of Illumina sequencing that targeted the V3-V4 hypervariable region of the 16S rRNA gene from all the collected samples revealed in total 25 OTUs present at an abundance of 1% or more in at least one of the colonies. OTUs were numbered in order of decreasing abundance, taking into account all the colonies. The first two OTUs accounted for 81% and the first 10 accounted for 95% of all the reads, considering all the colonies together. Dedicated phylogenetic trees constructed with our sequences from cultures and field samples and those downloaded from the NCBI database (Fig. 7) allowed us the taxonomic assignment of these OTUs, as previously carried out^27,28^.

Fourteen OTUs were mapped to the heterocystous subtree (Fig. 7a). The most abundant OTU (OTU1) fell into a very well-supported cluster with other *Rivularia* sequences. The percent identity between OTU1 and the other sequences in this cluster ranged from 97.6 to 100%, with the OTUs being 100% similar to an environmental colony collected from the Alharabe River in southwestern Spain^11^ and to other environmental samples isolated from the Baltic Sea^29^. Thus, OTU1 was assigned to *Rivularia* sp. OTUs 2 and 13 also fell in a very well-supported cluster with *Cyanomargarita melechinii* and *Cyanomargarita calcarea*. OTU2 was more similar *to C. melechinii* (99.52%), while OTU13 was more similar to *C. calcarea* (98.07%). Both OTUs were assigned to *Cyanomargarita* sp. OTUs 3, 11, 14, and 26 clustered with other *Calothrix* sequences; thus, they were assigned to this genus. OTUs 20 and 4 were included in a cluster with *Macrochaete* strains and were assigned to this genus; OTU4 was 98.3% similar to *Macrochaete lichenoides*, and OTU20 was 96.4–96.63% similar to *Macrochaete psychrophila*. OTU6 was mapped to a cluster that included other *Calothrix* sequences and *Scytonematopsis contorta* strains, with similarities ranging from 95.9 to 98.3%, as well as other environmental sequences obtained from *Dichothrix* tufts from the Muga River, which were 99.5% similar; therefore, it was assigned to *Dichothrix* sp. OTU8 was assigned to *Scytonema* sp. since it mapped to a well-supported clade with other *Scytonema* sequences. OTU19 clustered with other sequences of *Nostoc* and was assigned to this genus, while OTU15 was mapped to a sister clade of *Anabaena*, *Nostoc* and *Tolypothrix* strains and was therefore assigned only to the *Nostocaceae* family. Finally, OTU9 mapped to a cluster with an uncultured bacterium, and the maximum similarities found in the NCBI database were 95.22 with an uncultured bacterium and 93.78 with *Calothrix* sp. CAL3363^29^; therefore, this OTU was assigned to the *Nostocales* order.

Regarding the nonheterocystous cyanobacteria, eleven OTUs mapped to this subtree (Fig. 7b). The most abundant OTUs, OTU5 and OTU7, fell in two well-supported clusters with *Oculatella* strains and *Phormidium* strains, respectively. OTUs 16, 17, 18, 24 and 27, assigned to *Leptolyngbya* sp., mapped to clades with other *Leptolyngbya* sequences. OTU28 clustered with other *Schizothrix* strains. OTU10 and OTU21 could be assigned only to Leptolyngbyaceae and Oscillatoriales, respectively, due to their low percent identity with other sequences in the databases. Finally, OTU12 remained unassigned since no match was found in the databases for this sequence, and there was no clustering with any close relatives in the phylogenetic tree (Fig. 7b).

### Phylotype diversity in individual colonies

**Figure 8:**
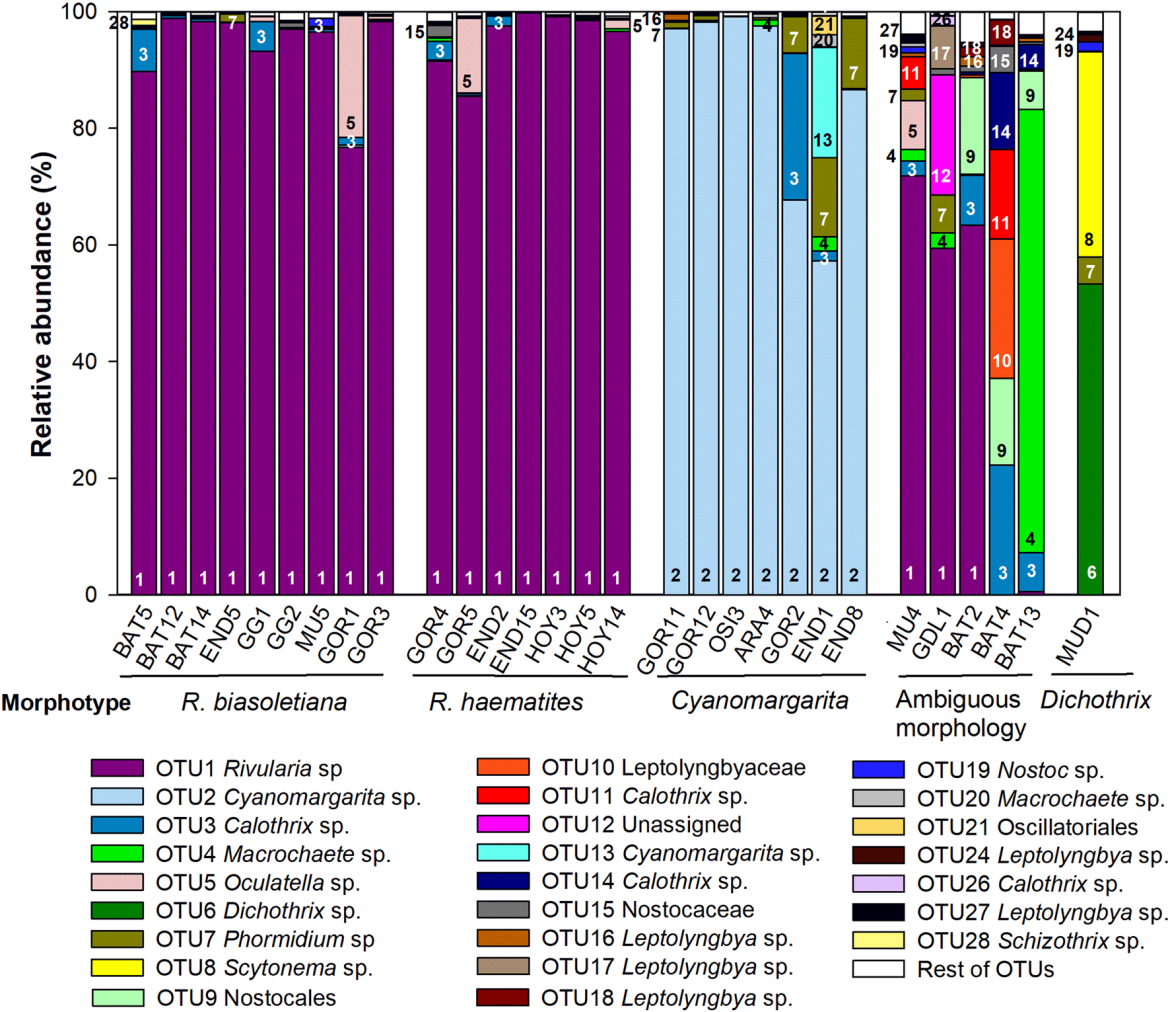
Phylotype diversity in individual colonies. The operational taxonomic units (OTUs) are represented by different colors and corresponding numbers. The colors correspond to those in Fig. 7. See Table 1 for the names and geographical origins of the environmental samples.

Both types of identified *Rivularia* colonies, *R. biasolettiana* and *R. haematites,* showed a clear dominance of OTU1 (Fig. 8). In six of the nine colonies identified as *R. biasolettiana*, OTU1 presented abundances from 97 to 99%, and in three, this proportion decreased to 76.8–93%. In six of the seven colonies of the *R. haematites* type, the abundance of OTU1 ranged from 97 to 99.8%, and in one colony, the abundance was 85.5% (Fig. 8). OTU3, corresponding to *Calothrix* sp., and OTU5, corresponding to *Oculatella* sp., were other phylotypes found in these colonies, with abundances ranging from 1.4–8.6% for *Calothrix* and 1.5–20.9% for *Oculatella* (Fig. 8).

In the seven *Cyanomargarita* colonies, OTU2 dominated, ranging from 97.1 to 99.2% in 4 colonies, decreasing to 86.6% in another colony, and further decreasing to 67.6 and 57.28% in the other two colonies. Regarding the other phylotypes found in these colonies, OTU7, corresponding to *Phormidium* sp., was the most abundant and present in all the colonies, with abundances of 12% and 13.5% in two of the colonies (Fig. 8). A phylotype corresponding to *Calothrix* sp., found in previously analyzed *Rivularia* colonies (OTU3), was also found in a colony of *Cyanomargarita* (GOR2), with an abundance of 25%. OTU13, which corresponded to another phylotype of *Cyanomargarita*, was present in only one colony (END1), with a 13.5% abundance.

The four *Rivularia*-like colonies with ambiguous morphologies were the most variable in terms of phylotype composition (Fig. 8). In the MU4, GDL1 and BAT2 colonies, *Rivularia* sp. (OTU1) predominated, although at different proportions. In the MU4 colony, OTU1 accounted for 72% of the total abundance, but *Oculatella* sp. (OTU5, 8.37%) and *Calothrix* spp. (OTU11 and OTU3, 8% overall) coexisted. In GDL1, the abundance of OTU1 was 59.4%, and that of unassigned OTU12 was 20.6%, which corresponded to the large proportion of other filaments observed under the microscope (Fig. 4e,g). This colony also contained *Leptolyngbya* sp. (OTU17), with an abundance of 7.3%, and *Phormidium* sp. (OTU7), with an abundance of 6.6%. The BAT2 colony, in addition to 63.4% OTU1, contained 8.6% *Calothrix* sp. (OTU3) and 16.5% Nostocales OTU9 (Fig. 8).

Despite its macroscopic similarity to the *Rivularia* colonies and clear banded appearance (Fig. 4d,f), BAT4 presented a negligible abundance of OTU1 (0.06%). This colony was composed of several OTUs at different proportions, such as *Calothrix* phylotypes corresponding to OTUs 3, 11 and 14 (22.2%, 15.3%, and 13.2%, respectively), Nostocales OTU9 (15%), Leptolyngbyaceae OTU10 (24%), and, to a lesser extent, Nostocaceae OTU15 (4.5%) and *Leptolyngbya* OTU18 (4.3%) (Fig. 8).

The *Rivularia*-like BAT13 colony also presented a negligible abundance of OTU1 (0.6%) and was dominated by OTU4 (76%), which was phylogenetically similar to the new genus *Macrochaete.* This colony also harbored, albeit to a lesser extent, OTUs 3 and 14, corresponding to *Calothrix* (6.7% and 4.2%, respectively), and Nostocales OTU9 (6.6%) (Fig. 8).

The MuD1 tuft was composed mainly (53.2%) of OTU6, assigned to *Dichothrix* sp., followed by OTU8 (*Scytonema* sp., 35.2%), OTU7 (*Phormidium* sp, 4.6%), and at a small proportion, OTU19 (*Nostoc* sp., 1.6%) and OTU24 (*Leptolyngbya* sp., 1.2%) (Fig. 8).

## Discussion

### Genotypic heterogeneity in single Rivularia-like colonies

*Rivularia*-like colonies have a global distribution, occurring in marine or freshwater habitats, where they are usually attached to a rocky substrate; however, many studies have reported that *Rivularia* spp. are associated with unpolluted environments^14^. In addition, the relationships between some morphological or physiological features and the environment make these species excellent environmental indicators of changes in running water quality, mainly related to eutrophication processes^14,30^. Therefore, they have been included in biomonitoring programs^21,31,32^. On the other hand, because *Rivularia* colonies sometimes persist for very long periods, avoiding grazing, the toxicity of these colonies is being investigated^33^. It is undoubted that in all of these approaches, where genera and species must be strictly identified from environmental samples, accurate cyanobacterial characterization is essential.

Traditional identification of cyanobacteria involves assigning a colony to a morphospecies, and conventionally, a bacterial colony is defined as a visible mass of clonal microorganisms, all of which originated from a single cell. However, the results from the present study show that the majority of the analyzed colonies consist of different clones growing together. Among the 28 *Rivularia*-like colonies, the phylotype corresponding to *Rivularia* sp. was present in 19 colonies, with abundances ranging from 59.4 to 99.8% depending on the studied colony. Nevertheless, it should also be noted that in most of the colonies, this phylotype dominated, whereby in 14 colonies, it presented an abundance of ≥ 90% (and within 7 of these colonies, the abundance was close to 99%). However, in three colonies, the abundance ranged from 72 to 85%, and in two of them, the abundance decreased to approximately 60%. The other highly abundant phylotypes found in these colonies, which reached abundances up to approximately 21%, corresponded to *Calothrix* sp. and *Oculatella* sp., the latter a genus morphologically similar to *Leptolyngbya* but separated from it because of genetic differences^34^. These results indicated great variability in the abundance of the phylotype corresponding to *Rivularia* depending on the analyzed colony, as well as variation in the other phylotypes and their abundances found in these colonies.

One of the surprising findings was that among the twenty-eight analyzed *Rivularia*-like colonies, seven corresponded to the new, recently described genus *Cyanomargarita*, which as the authors described, is virtually indistinguishable from *Rivularia* in field samples^15^. In these colonies, genotypic heterogeneity was also found, in which the abundance of the phylotype corresponding to *Cyanomargarita* varied from 57,28% in a colony with clear lamination resembling *R. haematites* (see Fig. 3b) to 99.2% in a soft colony resembling *R. biasolettiana*. Interestingly, in these colonies, *Phormidium* sp. was the dominant nonheterocystous cyanobacterium instead of *Oculatella* from *Rivularia* colonies, but the phylotype corresponding to *Calothrix* was also found.

Furthermore, phylotypes corresponding to *Cyanomargarita* and *Rivularia* were never found together in the same colony, although both types of colonies coexisted in the same rivers (e.g., Gordale Beck and Endrinales). Allelopathic effects could explain these results, as previously suggested for other cyanobacteria^35^. In fact, García-Espín et al.^33^ showed that extracts obtained from *Rivularia* colonies affected the photosynthetic activity of several diatoms and a red alga. Further experiments with extracts from both colonies would confirm this possible effect.

Another very surprising finding was that two *Rivularia*-like colonies did not present any phylotypes corresponding to *Rivularia* or *Cyanomargarita* (or contained them at an abundance ≤ 0.7%). In one of these colonies (colony BAT4), five different phylotypes were found at similar abundances (approximately 15–20%), of which three corresponded to different *Calothix* spp. and the others corresponded to other Nostocaceae and Leptolyngbyaceae. In the other colony (BAT13), the dominant phylotype corresponded to the new genus *Macrochaete*^16^. This genus has been described only from cultures, so to the best of our knowledge, this is the first report in which a natural population is morphologically and genetically characterized. Nevertheless, it is noteworthy that the morphological characteristics of filaments and trichomes in this environmental sample were different from those reported in the description of this new genus, in which the phenotypic features resembled those of *Calothrix*. However, these features corresponded only to isolated strains, which are known to exhibit morphological variability and differences from natural populations^7,12,13^.

### *R. biasolettiana* vs *R. haematites*

However, what was very interesting and deserves to be highlighted is that when we tried to differentiate the two typical *Rivularia* colonies found in calcareous streams, *R. biasolettiana* and *R. haematites*, we did not find genetic differences, at least at the studied level, the 16S rRNA gene.

16S rRNA is the most widely used marker gene^36,37^, which fits the criteria of ubiquity, regions of strong conservation, and regions of hypervariability^38,39^. This gene is supported by reference databases containing over a million full-length 16S rRNA sequences, therefore spanning a broad phylogenetic spectrum^40^. The 16S rRNA gene has served as the general framework and as the benchmark for the taxonomy of prokaryotes^41^. Advances in high-throughput sequencing technologies have enabled almost comprehensive descriptions of bacterial diversity through 16S rRNA gene amplicons, which have been used in surveys of microbial communities to characterize the composition of microorganisms present in environments worldwide^42–45^. Although some issues have been raised, such as identification of metabolic or other functional capabilities of microorganisms when studies focus only on this gene, recent studies have shown that the phylogenetic information contained in 16S marker gene sequences is sufficiently well correlated with genomic content to yield accurate predictions when related reference genomes are available^46–49^. Therefore, the 16S rRNA gene continues to be the mainstay of sequence-based bacterial analysis, vastly expanding our understanding of the microbial world^50^.

In particular, in cyanobacteria, as in other prokaryotes, the 16S rDNA gene is currently the most commonly used marker for molecular and phylogenetic studies^51,52^. The information obtained from 16S rDNA gene phylogenetic reconstructions, together with morphological, ultrastructural, and ecological data, led Komárek et al.^53^ to propose the current accepted classification of cyanobacteria. There have also been specific studies by this group concerning the problems associated with single-gene phylogenies, in which robust phylogenomic trees of cyanobacteria derived from multiple conserved proteins have also shown congruence between the multilocus and 16S rRNA gene phylogenies, which once again demonstrates the considerable strength of the 16S rRNA gene for phylogenetic inference and evaluation of prokaryote diversity^54–57^.

In this study, in contrast to the genetic identity found in *R. biasolettiana* and *R. haematites* colonies, showing a dominance of OTU1, the remainder of the studied representatives of Rivulariaceae showed a wide range of variation in the 16S rDNA sequences and with OTU1. Sequence identity between OTU1 and the remaining OTUs belonging to this family was as low as approximately 90%, ranging from 90.73 to 93.41%, and when it was compared with other Rivulariaceae from the databases, in the different clusters of the phylogenetic tree, this value ranged from 87.12 to 93.90%. A large difference between the sequences of this gene was also found in other studies on Rivulariaceae^15–17,29,58^. In fact, several new genera are emerging on the basis of these differences^15–17^. Comparisons of phylogenies using other markers, such as the phycocyanin operon and the intervening intergenic spacer (*cpc*BA-IGS) with the 16S rRNA gene in previous studies in Rivulariaceae, have shown largely consistent results, with a high level of divergence between the components of this family^11^.

In addition, the results of the present study showed correlations between morphological characteristics and the analyzed genes in all the cyanobacterial colonies/tufts, except for those of *R. biasolettiana* and *R. haematites*. In these two cyanobacteria, only distinct macroscopic phenotypic features were observed due to zonation and different degrees of calcification since no significant differences were found in the size measurements or other microscopic characteristics.

Therefore, although the remainder of the genome has not been studied in these populations, the genetic identity of the studied marker, phenotypic features, together with environmental preferences point out that *R. biasolettiana* and *R. haematites* are ecotypes of the same species, as previously suggested^59^.

*R. biasolettiana* and *R. haematites* have very similar morphotypes, and traditional taxonomical classification and studies have distinguished them primarily by their degrees of calcification. *R. biasolettiana*-type colonies are described as more gelatinous and less calcified, and the crystals are disseminated; however, *R. haematites* colonies are very hard and exhibit extensive calcification in concentric zones, which leads to clear lamination^24,25,60,61^. Because of its heavy mineralization, *R. haematites* is a model for stromatolite-binding organisms^25,26^.

Microscopic observations from this study showed that some colonies presented typical *R. haematites* morphology with concentric bands of intense calcification (see Fig. 2a,b), and others were soft and less calcified, such as *R. biasolettiana,* although all of them presented the same dominant phylotype. Many others with this dominant phylotype have also shown ambiguous morphology with no clear lamination, although some dark/light zones could be observed (see, e.g., Fig. 4b,d, f). Even in *Cyanomargarita* colonies, whose genotype was clearly separated from that of *Rivularia*, concentric zones and extensive calcification could be observed (see, e.g., Fig. 3b,d). These results suggested that these phenotypic features are not diagnostic characteristics for further identification.

In a two-year study, Obenlüneschloss and Schneider^61^ found that not all analyzed *R. haematites* colonies showed distinct concentric calcification layers. In the stromatolites of both types of *Rivularia,* the same lamination was observed, and the differences in calcification appeared later^60^. Pentecost and Franke^26^ compared populations of *R. biasolettiana* and *R. haematites* and argued that although both could be distinguished by their form of calcification and their trichome diameter, some populations of *R. biasolettiana* were more intensely calcified than others, suggesting that a continuity of forms may exist, even within the same stream, and therefore, a continuum of colony forms probably occurs between these taxa.

Differences in the calcification pattern have been attributed to seasonality and cyanobacterial activity, in particular to photosynthesis^24,26,62^. The calcification in *R. haematites* occurred in concentric bands, which varied in thickness and the density of crystals. Since characteristic zonation is formed by filaments of different successive generations, the thickness will vary depending on the growth rate, while crystal density will depend on the rate of calcification. Calcification is the result of photosynthesis (with a maximum of 14%) and evaporation during the warmer seasons, while it is entirely abiogenic during winter as a result of CO_2_ evasion^63^. Therefore, dense calcified bands similar to those formed in winter have been described that are caused by a reduction in trichome growth and EPS production, allowing the development of abiotic surface precipitate, and less calcified layers are formed during spring and summer, when calcification is associated with photosynthesis in zones of growth with cell division^24,26^. Thus, differences in climatic conditions and/or biological activity seem to lead to differences in the degrees of zonation and calcification.

The growth of *Rivularia* colonies is seasonal and strongly correlated with water temperature^24,26^. The colony growth rates were 12–14 µm/day in summer and 2 µm/day in winter^24^. The occurrence of *R. biasolettiana* was more closely related to high temperatures than that of *R. haematites*^21^. Moreover, colonies of *R. haematites* were generally collected under temperatures below 15 °C in mountain running waters^64^, and *R. haematites* stromatolites have been described as preferentially developed in wet periods, particularly in autumn and winter^60^. Our own field observations during the sampling for this and previous studies were that the gelatinous and weakly calcified *R. biasolettiana* type was more abundant in warmer locations, and in contrast, *R. haematites* was dominant in cold locations (data not shown).

One possible explanation for the results found in this study could be related to these differences in the degree of zonation and calcification in relation to climate, which could include microclimatic conditions. In warmer sites or climatic conditions, when growth is rapid, the number of filaments will increase, moving towards the surface in a weakly dense and unaligned arrangement, on which calcite crystals spread, providing a lighter and less calcified structure. Thus, increased growth of *Rivularia* colonies can lead to the *R. biasolettiana* type. Under colder conditions, such as in winter, or microclimatic conditions, when growth slows down for other reasons, such as low light, filaments become more densely packed, allowing the development of extensive precipitates and leading to a dark band. When these conditions change, e.g., in the spring and summer, increases in temperatures and/or light will result in increasing and faster growth, leading to a less calcified new layer, and successive seasonal and/or microenvironmental changes will result in the typical lamination of *R. haematites*. Therefore, warmer places with high temperatures and/or light will allow the occurrence of the *R. biasolettiana* type, while in colder sites and/or sites with alternating environmental conditions, the *R. haematites* type will develop. Shaded colonies and colonies that lie in the supratidal spraywater zone often contain small, irregular and more densely packed crystals^61^.

Cyanobacteria are known to modify EPS production, pigments, and morphology under environmental stimuli^6^. The production of EPS also varies depending on the cyanobacteria, whereby *Rivularia* has shown a well-developed exopolymer layer^65^, which is of great importance for this epilithic cyanobacteria, as it acts as an adhesive that allows cells to stick to the stones in the running waters, and it holds the filaments together, minimizing cell damage during intermittent drying exposure to the air and evaporation in the warmer seasons^66^. The C/N ratio is an important parameter for the variation in EPS production since high amounts of fixed C compared to N levels drive EPS synthesis to store excess C^67,68^. Therefore, *Rivularia* colonies that are exposed, in spring and summer, to high light intensities and temperatures will increase their photosynthetic rates and therefore the amount of EPS, as shown by the *R. biasolettiana* morphotype. In addition, most of the analyzed populations were dark in color, probably in relation to the accumulation of the yellow–brown scytonemin pigment in the sheaths or EPS, as previously observed in shallow and clear oligotrophic ecosystems, where water clarity allows UV radiation to penetrate well, protecting the cells from the damaging effects of this radiation^69,70^.

In conclusion, environmental factors can lead to differences in macroscopic phenotypic features, such as those found in the *Rivularia* colonies studied here. However, further sampling under different climatic conditions and/or microenvironmental conditions or of *Rivularia* cultures grown under distinct temperature and/or illumination conditions, as well as analysis of other genes, are needed to confirm this hypothesis.

## Methods

### Sampling

The locations of the sampling sites from which samples were collected and their codes are shown in Table 1. All the sampled rivers or streams were characterized by highly calcareous waters. The Muga, Guarga, Osia, and Arás rivers are located in northeastern Spain, the Hoyas and Endrinales streams and Bogarra River are located in southeastern Spain, and the Guadiela River is located in central Spain. The Gordale Beck sampling site was located near Malham, West Yorkshire, northern England, from which *Rivularia haematites* has been reported^24,26^. From these locations, twenty-one *Rivularia*-like colonies were sampled in Spain, and seven in the United Kingdom. In addition, a *Dichothrix* tuft was collected from the Muga River, Spain. The samples were kept cold until reaching the laboratory, where they were divided into two parts, after washing them and checking by microscopy that there were no cyanobacterial contaminants outside the colonies. One part from each sample was used for morphological characterization and the other frozen and stored at − 20 °C until DNA extraction for genetic characterization. The colonies or tuft were named after the river or stream where they were collected, followed by a number (Table 1).

### Morphological characterization

Samples were inspected under a Leica MZ12.5 dissecting stereomicroscope (Leica, Leica Microsystems, Wetzler, Germany) equipped with epifluorescence and video camera systems and by an Olympus BH2-RFCA photomicroscope (Olympus, Tokyo, Japan) equipped with phase-contrast, epifluorescence, and video camera systems (Leica DC Camera, Leica Microsystems). To better observe the samples via microscopy, several colonies were decalcified with 0.5 M EDTA. Representative colonies of *Rivularia*-like samples were analyzed, except those with ambiguous morphologies, for which the variability of the morphotypes found within the colony precluded analysis. Size measurements were analyzed using SigmaScan Pro 5.0.0 software. Kruskal–Wallis one-way analysis of variance of ranks was used to determine the significance of differences between size measurements.

### Genetic characterization

Genomic DNA was extracted from individual colonies using a Power Biofilm DNA Extraction Kit (Mo Bio, Carlsbad, CA, United States) following the manufacturer’s instructions, with a modification at the beginning of the protocol as previously described^28^ to improve the lysis of the cells.

The phylotype composition of the cyanobacterial colonies was assessed by amplicon metagenomics targeting the hypervariable V3-V4 region of the 16S rRNA gene, using an Illumina MiSeq sequencer (Illumina Inc., San Diego, CA, USA) at the Genomic Service of the Universidad Autónoma de Madrid. First, PCR was performed using the cyanobacterial-specific primers CYA359F and 781Ra/781Rb^71^ in separate reactions for each reverse primer as suggested by Boutte et al.^72^ plus a targeted sequence. PCR was carried out with 1 ng of DNA, Q5 Hot Start High-Fidelity DNA Polymerase (New England Biolabs), and 100 nM primers in a 25 μl volume, and the cycling conditions were 1 × 98 °C for 30 s, 23 × 98 °C for 10 s, 54 °C for 20 s, and 72 °C for 20 s, and 1 × 72 °C for 2 min. The second PCR using barcoded primers for each colony was performed in a 20 μl final volume using the same DNA polymerase, 1 μl of the first PCR product, and 400 nM primers with the following cycling conditions: 1 × 98 °C for 30 s, 7 × 98 °C for 10 s, 60 °C for 20 s, and 72 °C for 20 s, and 1 × 72 °C for 2 min.

PCR products were quantified, pooled in equimolar amounts and purified using AMPure Beads (Beckman Coulter) prior to sequencing with a MiSeq sequencer (Illumina Inc., San Diego, CA, USA) at a read length of 2 × 300 bp. At least 100,000 sequences were obtained for each amplicon.

Sequence data were processed using QIIME v.1.9.0^73^ as previously described^28^. The first taxonomical assignment of the OTUs was performed against the Greengenes database^74^ by the classifier method of the Ribosomal Database Project with a confidence value of 0.8^75^, which allowed the removal of OTUs identified as chloroplasts or noncyanobacterial organisms. Afterwards, OTUs were matched against our sequence dataset of isolated cultures and field samples by employing the ‘uparse ref command’ in USEARCH^76^. In addition, a previously sampled *Dichothrix* field tuft from the Muga River was analyzed by cloning almost the entire 16S rRNA gene, as previously described^16^. Then, all this information was compared with the taxonomic assignments made against the Greengenes database^74^ as mentioned above and against the SilvaMod database^77^ using the lowest common ancestor (LCA) algorithm implemented in CREST^78^. In addition, the representative OTU sequences were compared with those in the NCBI database using a BLAST search (https://www.ncbi.nlm.nih.gov/blast), and sequences similar to them were downloaded to construct phylogenetic trees as previously described^27,28^. The first tree was constructed with only full-length 16S rDNA sequences (approximately 1500 bp), which were aligned using ClustalW in BioEdit 7.0.5.3^79^. After this, the OTU sequences (approximately 420 bp) were aligned against the alignment of the first tree, and a new tree was constructed and compared with the previous tree to ensure that the clades were maintained. Phylogenetic trees were computed using the neighbor-joining method^80^ in Mega 7^81^ with 1000 bootstrap replicates. Evolutionary distances were computed using the Tajima-Nei method^82^, and pairwise deletion was used to account for sequence-length variation and gaps. The percent identity between sequences was determined as (1-p-distance)*100.

Because OTU1 appeared at markedly high abundance, amplicon sequence variant (ASV) determination was performed using the DADA2 pipeline in QIIME 2^83^, showing that ASVs assigned to this OTU differed by only one nucleotide and were therefore indistinctly distributed between the different samples.

Alpha diversity indices (Chao1 estimator, Good’s coverage and observed OTUs) were calculated using QIIME. Good’s coverage estimates reached 100% in all the samples, indicating that the large majority of the cyanobacterial diversity was captured.

The OTU sequences have been deposited in the GenBank database under accession numbers MT335702-MT335726. Raw sequencing data have been deposited in the NCBI Sequencing Read Archive under accession number PRJNA648107.
